# Correlation Between Clinical and Histopathological Stagings of Oral Submucous Fibrosis: A Clinicopathological Cognizance of 238 Cases From South India

**DOI:** 10.7759/cureus.49107

**Published:** 2023-11-20

**Authors:** Deepak Pandiar, Priyadharshini G, Reshma Poothakulath Krishnan, Uma Maheswari T N

**Affiliations:** 1 Oral Pathology and Microbiology, Saveetha Dental College and Hospitals, Saveetha Institute of Medical and Technical Sciences, Saveetha University, Chennai, IND; 2 Oral Medicine and Radiology, Saveetha Dental College and Hospitals, Saveetha Institute of Medical and Technical Sciences, Saveetha University, Chennai, IND

**Keywords:** staging, oral submucous fibrosis, malignant transformation rate, histological, dysplasia, clinical

## Abstract

Background

This paper aims to descriptively present the clinico-demographic and pathological profile of 238 cases of oral submucous fibrosis (OSMF) with emphasis on the correlation between clinical and histopathological classification systems and the incidence of malignant transformation.

Methods

A total of 7098 oral biopsies were retrospectively retrieved over a period of 13 years, out of which 238 cases of OSMF were included in the present study. Data were analyzed for age, gender, habits, clinical symptoms, functional staging, histological staging, type and nature of epithelium, signet-ring cell changes, presence/absence of dysplasia or transformation squamous cell carcinoma, and treatment.

Results

Clinically and histologically, most cases were moderately advanced. Men outnumbered women. The prevalence of dysplasia was found to be 23.94% and the malignant transformation rate was estimated to be 13.8%. There was a significant correlation between clinical staging with age and histological grading. No correlation was found between histological staging and the age of the patients.

Conclusions

OSMF is a habit-related progressive chronic oral condition with a high malignant transformation rate. Multimodal treatment with auxiliary physiotherapy helps in the improvement of the clinical symptoms.

## Introduction

Oral submucous fibrosis (OSMF) is a progressive, habit-related, insidious oral potentially malignant disorder and one of the common oral health problems in Southeast Asia, especially in the Indian subcontinent [1-3]. The prevalence of OSMF in India ranges from 0.2-2.3% and 1.2 -4.6% in males and females, respectively [4]. There are various etiological factors leading to the development of OSMF, including areca nuts, tobacco products, spicy foods, vitamin B-complex deficiency, and even genetic and immunologic processes. Areca nut chewing with slaked lime and tobacco are the main etiological factors for the development of OSMF; the slaked lime increases the absorption of the alkaloids released from the areca nut [5]. Arecoline, arecaidine, guvacoline, and guvacine are the main alkaloids contained in the areca nut [6]. A number of factors collectively increase the cross-linking of collagen and the proliferation of fibroblasts. These include nitrosation of arecoline and subsequent inflammatory reaction, which further activates tissue inhibitors of matrix metalloproteinases (TIMPs) and the copper-mediated activation of lysyl oxidases [7]. Altogether the aforementioned factors cause fibrosis of the lamina propria leading to hyalinization and epithelial atrophy [8]. 

Arecoline has cytotoxic effects, which cause specific variations in the regulatory proteins of the cell cycle [9]. This leads to endothelial cell damage and ultimately reduced vascularity. According to the traditional theory of the pathogenesis of OSMF, the degree of vascularity decreases as the pathological stage advances. Reduced vascularity and extensive fibrosis deny systemic absorption of carcinogens and have a negative effect on the atrophic compromised epithelium, resulting in malignization [2]. In literature, the overall malignant transformation rate (MTR) of 4.2% and 6% has been estimated independently in recent systematic reviews [10-11].

Despite being a common condition of the oral cavity with a high malignant transformation rate, there is limited data on descriptive clinicopathological studies, the presence of dysplasia, and the malignization of OSMF. The present paper aims to descriptively present the clinico-demographic and pathological profile of 238 cases of OSMF from a single tertiary oral health care center in Chennai, India, with emphasis on the correlation between clinical and histopathological classification systems and the incidence of malignant transformation.

## Materials and methods

The present retrospective study included data from 7098 oral biopsies from the archival files (including the institutional electronic database) of our institution, a tertiary oral health care center from Chennai, India, over a period of 13 years (2009-2022). The study was conducted in Saveetha Dental College and Hospitals, Chennai, India, after ethical approval was granted by Saveetha Dental College-Institutional Human Ethical Committee (SDC-IHEC) with approval number IHEC/SDC/FACULTY/23/OPATH/263. Two hundred and thirty-eight cases diagnosed as oral submucous fibrosis were included in the present study after satisfying the inclusion criteria. All the cases that were histopathologically confirmed on incisional biopsy and had complete clinical and demographic data along with H&E stained slides or formalin-fixed paraffin-embedded (FFPE) blocks were included in the present study. Cases without slides or FFPE blocks and other collagen-related systemic diseases or conditions leading to restricted mouth opening were excluded. Data were analyzed for age, gender, habits, clinical symptoms, functional staging, histological staging, type and nature of epithelium, signet ring cell changes, presence/absence of dysplasia or transformation squamous cell carcinoma, and treatment. 

The cases were functionally staged based on the Khanna and Andrade, 1995, proposal as follows: a) Group I: Very early cases with mouth opening more than 36 mm; b) Group II: Early cases with mouth opening between 26-35 mm; c) Group III: Moderately advanced cases with mouth opening between 15-25 mm; d) Group IVa: Advanced cases with mouth opening 2-15 mm and e) Group IVb: Advanced cases with premalignant changes and malignant transformation [12]. 

All the slides were retrieved and reassessed regarding staining quality and readability. Two individuals observed the slides using the histological criteria put forward by Pindborg and Sirsat to histologically grade the cases as follows: Stage I: very early OSMF; Stage II: Early OSMF; Stage III: Moderately advanced OSMF and Stage IV: Advanced OSMF. The histological staging was done based on the nature of collagen, fibroblastic response, status of capillaries, and inflammatory response [7]. 

The data were entered in a Microsoft Excel 2021 spreadsheet (Microsoft Corporation, Redmond, Washington, United States) and finally, a database was generated using the IBM SPSS Statistics for Windows, Version 26.0 (Released 2019; IBM Corp., Armonk, New York, United States) software. Descriptive statistics was used for frequency counts. Statistical analysis was done using the Chi-square test and a p-value below 0.05 was considered significant statistically. 

## Results

Clinico-demographic profile

Biopsies of OSMF constituted 3.35% of all oral biopsies from January 2009 to December 2022. The mean age of occurrence was 40.94±12.35 years (median 39.00 years; range 18-77 years). The distribution of patients in decades of life is shown in Table 1.

**Table 1:** Detailed demographic profile of 238 cases of oral submucous fibrosis

Variable	Number (%)
Age (mean 40.94±12.35 years)	
Less than 19	2 (0.84%)
20-29	48 (20.17%)
30-39	71 (29.83%)
40-49	58 (24.37%)
50-59	37 (15.55%)
More than 60	22 (9.24%)
Gender (M:F::9.82:1)	
Male	216 (90.76%)
Female	22 (9.24)
Functional Staging	
I	11 (4.6%)
II	67 (28.2%)
III	105 (44.1%)
IV	46 (19.3%)
V	9 (3.8%)
Histological Staging	
I	2 (0.8%)
II	19 (8.0%)
III	105 (44.1%)
IV	112 (47.1%)
Keratinization	
Hyperparakeratinization	147 (61.8%)
Hyperorthokeratinization	68 (28.6%)
Both	7 (2.9%)
Non-keratinized	16 (6.7%)
Epithelial thickness	
Normal	2 (0.8%)
Hyperplastic	85 (35.7%)
Atrophic	151 (63.4%)
Dysplasia	
None	181 (76.1%)
Mild	21 (8.8 %)
Moderate	21 (8.8 %)
Severe	15 (6.3%)

**Figure 1:**
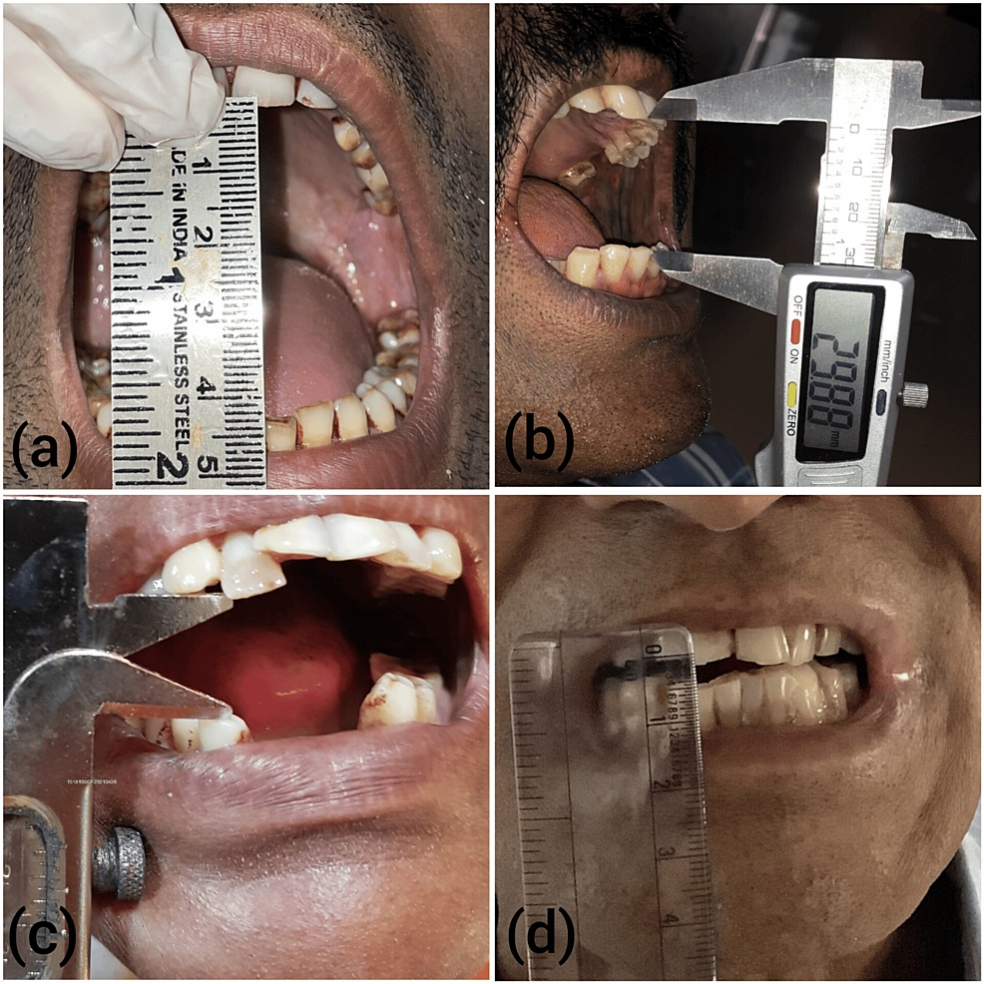
Clinical pictures demonstrating various clinical stages of oral submucous fibrosis. Note mouth opening as the disease progresses

The maximum number of cases was seen in the fourth decade (71/238) followed by the th decade; only 2 cases were seen at age younger than 19 years. Males clearly outnumbered the female gender with a ratio of 9.82:1 (216M:22F). In 186 case sheets, the details of associated deleterious habit was mentioned. The habit of paan chewing was predominantly associated with the disease (75/186) followed by synergistic use of paan and smoking (28/186). Areca nut usage, a known causative agent and risk factor, was reported only in 11 patients. Uncommon habits were the usage of hans, betel chewing, and alcohol consumption. Clinically, 44.1% of the cases (105/238) were in stage III, 28.2% were in stage II, and 19.3% of OSMF cases constituted stage IVa. In total, 11/238 and 9/238 were in stages I and IVb, respectively (Figure 1). All 9 cases of stage IVb showed a white non-scrapable patch (oral leukoplakia). 

Histopathological Characteristics

**Figure 2:**
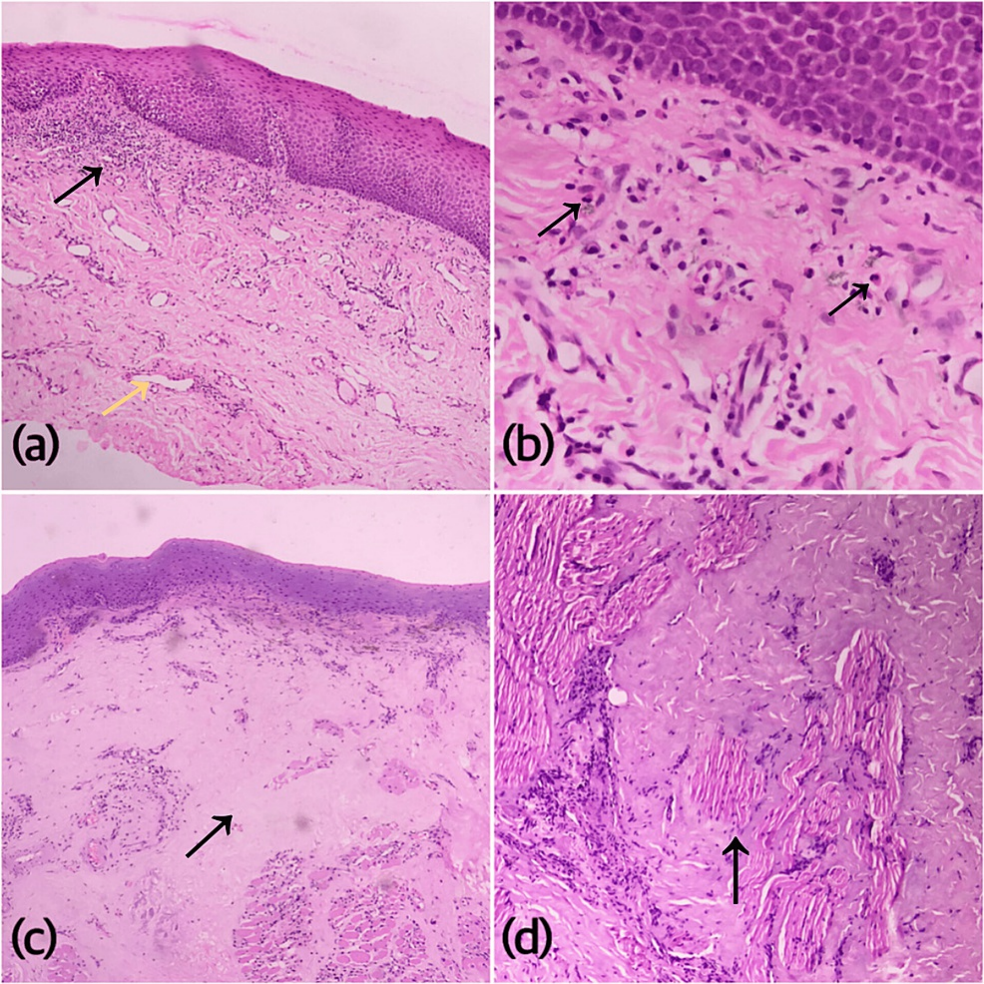
Photomicrographs of H&E stained sections showing a) early stage of OSMF with early hyalinization, numerous dilated vessels (yellow arrow), and mixed intense inflammation (black arrow) (40X), b) same case showing abundant eosinophils sub-epithelially (400X, black arrows), c) advanced OSMF with extreme hyalinization and homogenization of collagen with epithelial atrophy (40X, black arrow), and d) hyalinization in an advanced case of OSMF replacing skeletal muscle bundles (400X, black arrow).

Histologically, 44.1% (105/238) of the cases were reported as moderately advanced OSMF, and 47.1% (112/238) cases were categorized under advanced OSMF. There were only two cases of very early OSMF and the remaining 19 cases (8%) were early OSMF histopathologically (Figure 2).

**Figure 3:**
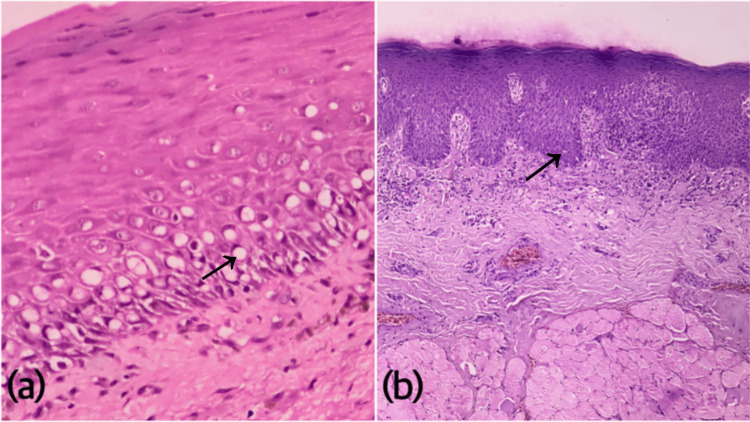
Photomicrographs of H&E-stained sections showing a) signet-ring cell changes predominantly seen in the basal and supra-basal layers (400X, black arrow) and b) moderate epithelial dysplasia in OSMF (100X, black arrow)

A total of 63.4% of the cases displayed atrophied epithelium (151/238); 93.3% of the cases showed hyperkeratosis, specifically parakeratosis (147/238) followed by orthokeratosis (68/238) and mixed ortho- and parakeratosis was noted in the remaining seven cases. Only 6.7% of the cases showed non-keratinized epithelium. Among the cases, which showed hyperkeratosis, an additional feature was noted in the epithelium, traditionally explained in literature as signet-ring-cell-like changes (99/238, 41.6%) (Figure 3a). Interestingly, these changes were found only in keratinized oral mucosa. The prevalence of dysplasia was found to be 23.94% (57 cases showed epithelial dysplasia; mild in 21 cases, moderate in another 21 cases, and severe dysplasia in the remaining 15 cases. Only two cases were categorized as very early OSMF and the remaining 19 (8%) were early OSMF histopathologically. A total of 63.4% of the cases displayed atrophied epithelium (151/238). Most cases showed hyperkeratosis (93.3%), specifically parakeratosis (147/238) followed by orthokeratosis (68/238) and mixed ortho- and parakeratosis (7/238). Only 6.7% of the cases showed non-keratinized epithelium. Among the cases which showed hyperkeratosis, an additional feature was noted in the epithelium, traditionally explained in literature as, signet-ring-cell-like changes (99/238, 41.6%) (Figure 3a). Interestingly, these changes were found only in keratinized oral mucosa. The prevalence of dysplasia was found to be 23.94% (57 cases showed epithelial dysplasia; mild in 21 cases, moderate in another 21 cases, and severe dysplasia in the remaining 15 cases (Figure 3b). 

Correlation of patients' ages with functional and histological stagings

Age-wise distribution (in decades of life) and its correlation with functional and histological stagings are demonstrated in Table 2 and Table 3, respectively.

**Table 2:** Correlation of functional staging of OSMF with age * statistically significant OSMF: oral submucous fibrosis

Age (in decades, years) Functional stage	I	II	III	IV	V	p-value: 0.009*
Less than 19	0	1	1	0	0	
20-29	3	16	20	9	0	
30-39	1	23	27	18	2	
40-49	3	15	29	9	2	
50-59	3	9	15	5	5	
More than 60	1	3	13	5	0	

**Table 3:** Correlation of histological staging of OSMF with age * statistically not significant OSMF: oral submucous fibrosis

Age (in decades, years) Histological stage	I	II	III	IV	p-value: 0.948*
Less than 19	0	0	1	1	
20-29	0	5	20	23	
30-39	0	4	32	35	
40-49	2	6	23	27	
50-59	0	2	18	17	
More than 60	0	2	11	9	

A significant correlation was found between the age of the patients and the functional staging (p-value 0.009), however, there was no significant correlation between the histopathological staging of OSMF and age (p-value 0.948). Further, a positive correlation was also found between the functional and histological stagings (p-value 0.009) (Table 4).

**Table 4:** Correlation of functional staging and histological staging of OSMF * statistically significant OSMF: oral submucous fibrosis

Histological stage Functional stage	I	II	III	IV	p-value: 0.009*
I	1	7	1	2	
II	1	9	38	19	
III	0	2	53	50	
IV	0	1	12	33	
V	0	0	1	8	

Treatment and follow-up

**Figure 4:**
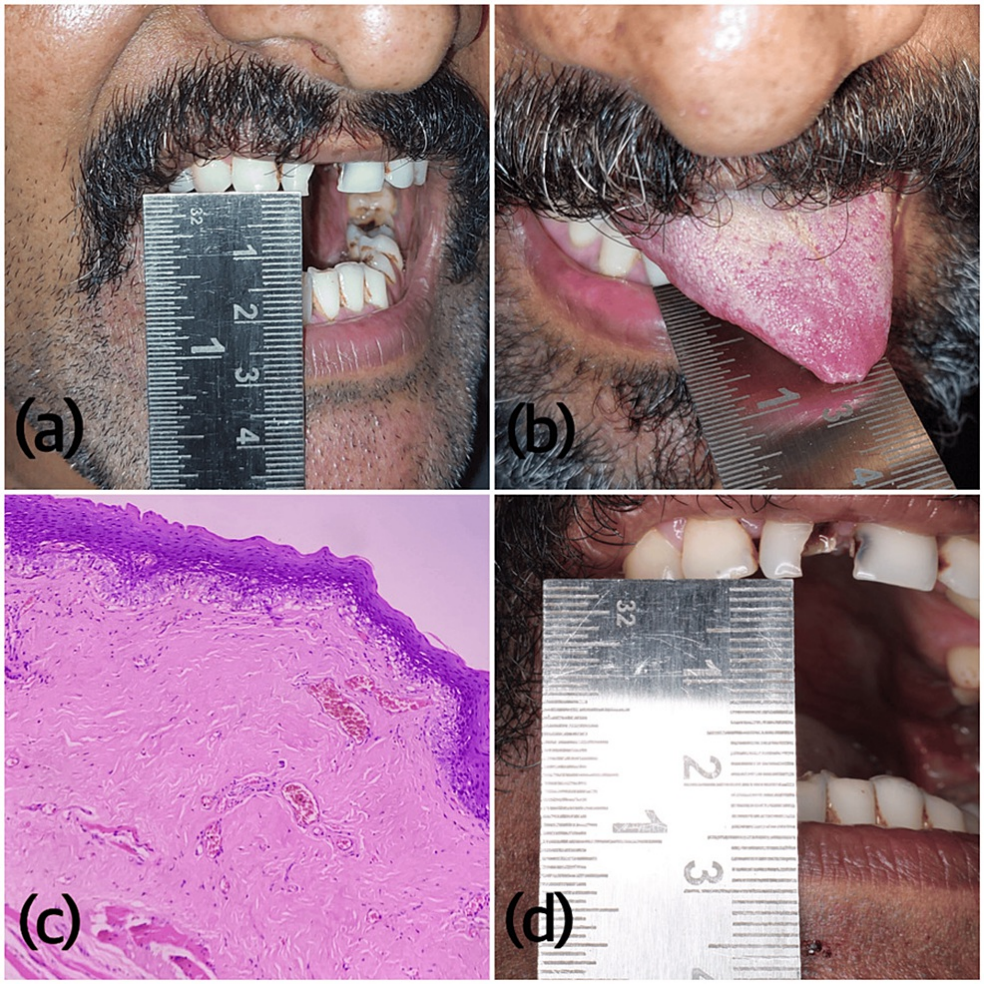
a) Clinical presentation showing restricted mouth opening with pale mucosae pre-treatment, b) The patient was unable to protrude the tongue entirely. The tip of the tongue was depapillated and the posterior part appeared coated, c) Photomicrograph of H&E-stained section showing hyalinization of the stroma with reduced vascularity and atrophied overlying mucosal epithelium (moderately advanced OSMF, 100X) and d) The clinical photograph showing post-therapeutic increased mouth opening OSMF: oral submucous fibrosis

For 167 patients, a detailed treatment plan was available. The standard protocol consisted of a prescription of lycopene (5000 mcg) capsule (once a day for two weeks) with supplementary iron. On subsequent visits, after one month, intralesional hyaluronidase (1500U), dexamethasone (4mg/ml), and 2% lignocaine were added, with continuation of lycopene. Additionally, physiotherapy using ice cream sticks was demonstrated and the patients were asked to follow it. Vitamin B12 was added to the regimen after another month. After ten intralesional injections, the mouth opening of all patients was assessed, and increased functionality was noted in all patients (increased mouth opening ranging from 2.5 mm to 9 mm). All the patients continued Vitamin B12 and iron supplements with continued physiotherapy, resulting in better functional outcomes (Figure 4). 

In addition to the 21 patients reported previously by us till 2021 [3], an additional 12 patients were reported to show malignant transformation. The malignant transformation rate in the current series was estimated to be 13.8%.

## Discussion

The present study included 238 cases of OSMF from a single tertiary oral health care center from Chennai with descriptive analysis of clinicopathological features. Oral submucous fibrosis is a progressive fibrotic disease that not only detrimentally affects the quality of life and function but also bears a very high MTR. Mainly affecting the natives of South-East Asian countries, it is now also reported in the immigrant Asian population of America and Britain [13]. The detailed analysis of clinicopathological features not only provides a baseline epidemiological data but also helps in understanding the geographic variation particularly pertaining to the habits and genetic predisposition. 

A peak incidence was noted in the fourth decade of life with a mean age of 40.94±12.35 years (median 39.00 years). The youngest patient was 18 years of age and the oldest patient was 77 years of age. The previous Indian studies have reported a similar distribution but in contrast, a lower age was reported in a Chinese cohort (35.23±10.08 years) [13]. Regardless, OSMF is not just a disease of adults. The youngest case of OSMF was reported in a four-year-old Indian girl in 1985 [14]. Similar cases are reported from India and adjacent countries in children who had a history of areca nut chewing or tobacco consumption [15,16]. Males clearly outnumbered the females, which is in concordance with the previously reported studies, irrespective of the region and country [13,17,18].

In the present study, consumption of paan chewing was predominantly associated with the disease followed by simultaneous usage of paan and smoking. Paan chewing and betel (areca) nuts with or without tobacco have previously been correlated positively with an increased risk of cancer in independent studies [19,20]. Also, it is a known fact that areca nut is a strong risk factor for OSMF. Areca nut, paan (±tobacco), and other commercially available products are not just addictive because of their euphoric effects but are also carcinogenic. In the present study, we found that about 93% of the cases demonstrated hyperkeratosis. Oral mucosa reacts differently to different stimuli. Hyperkeratosis is one such reaction to mechanical trauma to occlusion/occlusal forces or external stimuli, chewing habits in particular. It has been demonstrated that the expression of loricin, a late differentiation marker of terminally differentiated keratinocytes, is seen more in keratotic oral epithelial and OSMF as compared to normal epithelium, which further was shown to correlate with chewing habits [21]. Thus, hyperkeratosis in OSMF could be attributed to altered mechanisms of a cornified oral epithelial envelope. Friction-induced keratosis (internal or external stimuli) also causes interesting epithelial changes in OSMF, regarded as signet-ring cell change, which was noted in 41.6% of the cases, a percentage much higher than earlier studies where these changes were first mentioned (13-19.2%) [7,22]. However, the number of cases in our studies is also much higher. Mucosal erosion, attributed to mechanical trauma, and ischemia have been postulated to cause such changes in mucosal epithelium [22]. It's noteworthy that these changes should not be confused with koilocytic changes associated with viral etiopathogenesis.

Epithelial changes are the prime alterations seen in OSMF, which are either secondary to mechanical stimuli as mentioned before, or to the stromal changes such as hyalinization of collagen, collapsed blood vessels, or altered cytokine profile [2]. Epithelial atrophy is noted in OSMF as the stage advances; atrophic epithelium was noted in 63.4% of the cases in the present study. The systemic absorption of the carcinogens is denied aftermath of these changes, which further affects the already compromised epithelium, leading to dysplastic changes and finally to malignization. The prevalence of dysplasia was found to be 23.94%, but overall MTR in our cohort was 13.8%. A previous study from another South Indian region (Dharwad, Karnataka) reported a comparable malignant transformation rate of 11.6% from a cohort of 205 cases [18]. All the reported cases that showed malignant transformation were males. A male predilection corresponds to the number of cases showing OSMF. Here, the role of micronutrients, particularly copper, should not be overlooked, which possibly not only has a role in the progression of the disease but also could be responsible for a better grade of squamous cell carcinoma arising in the background of OSMF. All these cases were well to moderately differentiated and showed no extension to the deeper planes and no evidence of regional metastases.

The data pertaining to the correlation between functional or clinical and histopathological stagings is conflicting [17,23,24]. While most studies showed no correlation between the histological and clinical staging systems, we found a significant correlation between the histological and clinical staging of OSMF implying that clinically advanced OSMF shows extensive fibrosis and constricted vasculature histopathologically. The reason for no correlation could lie in the selection of the area for obtaining the incisional biopsy. In a study of 228 cases of oral submucous fibrosis, it was shown that the formation of bands initiates in the fauces, followed by the buccal and labial mucosa [25]. Universally, as the disease progresses, the mouth opening is restricted and the biopsy is taken from the more accessible anterior parts of the oral cavity; thus, histologically the disease may be at a lower grade; however, clinically it would have already been advanced, explaining the discordance. As stated in a study, the site of biopsy for diagnosis was the anterior buccal region [23]. In our study, the biopsy was taken from the most fibrotic areas of the buccal mucosa. We did not find any correlation between the age of the patient and histological staging implying that a histologically advanced disease may be seen in younger patients or vice versa; in contrast, functionally, there was a positive correlation with the age. As age advances, the severity of the disease worsens proportionately, depicting a progressive insidious course of the disease.

We here presented a large data of OSMF with detailed analysis of clinical parameters, histological features, and MTR; the results may however not be generalized to the whole population of Tamil ethnicity (an ethnicity indigenous to the South Indian state of Tamil Nadu). Also, many patients are lost to other hospitals which also affects the results. These are the inherent limitations of any hospital-based study. Still, the present study does provide baseline data for planning more descriptive intervention programs that would assist in the early diagnosis and management of OSMF.

## Conclusions

OSMF is a habit-related progressive chronic oral condition seen mainly in the natives of South Asian countries with a high MTR. The present study provides large robust data, with inherent limitations, pertaining to the prevalence and malignization of OSMF along with correlation with histological and clinical grading systems. Multimodal treatment with auxiliary physiotherapy helps in the improvement of the clinical symptoms. In most instances, the early clinical signs are non-specific and the patient is usually not aware of the disease. Mass education pertaining to the discontinuation of habits and early interceptive presentation to the physician may help in expeditious diagnosis and treatment.
